# Investigating the causal relationship and potential shared diagnostic genes between primary biliary cholangitis and systemic lupus erythematosus using bidirectional Mendelian randomization and transcriptomic analyses

**DOI:** 10.3389/fimmu.2024.1270401

**Published:** 2024-02-19

**Authors:** Tian Tao, Anqi Tang, Lizeyu Lv, Jianhua Yuan, Ling Wu, Liangbin Zhao, Jun Chen

**Affiliations:** ^1^ Department of Nephrology, Hospital of Chengdu University of Traditional Chinese Medicine, Chengdu, China; ^2^ Department of Cardiovascular Medicine, Chengdu Second People’s Hospital, Chengdu, Chengdu, China; ^3^ Department of Intensive Care Medicine, Hospital of Chengdu University of Traditional Chinese Medicine, Chengdu, China

**Keywords:** systemic lupus erythematosus, primary biliary cholangitis, Mendelian randomization, transcriptomic data, causal relationship

## Abstract

**Background:**

The co-occurrence of primary biliary cholangitis (PBC) and systemic lupus erythematosus (SLE) has been consistently reported in observational studies. Nevertheless, the underlying causal correlation between these two conditions still needs to be established.

**Methods:**

We performed a bidirectional two-sample Mendelian randomization (MR) study to assess their causal association. Five MR analysis methods were utilized for causal inference, with inverse-variance weighted (IVW) selected as the primary method. The Mendelian Randomization Pleiotropy RESidual Sum and Outlier (MR-PRESSO) and the IVW Radial method were applied to exclude outlying SNPs. To assess the robustness of the MR results, five sensitivity analyses were carried out. Multivariable MR (MVMR) analysis was also employed to evaluate the effect of possible confounders. In addition, we integrated transcriptomic data from PBC and SLE, employing Weighted Gene Co-expression Network Analysis (WGCNA) to explore shared genes between the two diseases. Then, we used Gene Ontology (GO) and Kyoto Encyclopedia of Genes and Genomes (KEGG) pathway enrichment methods to perform on the shared genes. The Least Absolute Shrinkage and Selection Operator (LASSO) regression algorithm was utilized to identify potential shared diagnostic genes. Finally, we verified the potential shared diagnostic genes in peripheral blood mononuclear cells (PBMCs)-specific cell populations of SLE patients by single-cell analysis.

**Results:**

Our MR study provided evidence that PBC had a causal relationship with SLE (IVW, OR: 1.347, 95% CI: 1.276 - 1.422, P < 0.001) after removing outliers (MR-PRESSO, rs35464393, rs3771317; IVW Radial, rs11065987, rs12924729, rs3745516). Conversely, SLE also had a causal association with PBC (IVW, OR: 1.225, 95% CI: 1.141 - 1.315, P < 0.001) after outlier correction (MR-PRESSO, rs11065987, rs3763295, rs7774434; IVW Radial, rs2297067). Sensitivity analyses confirmed the robustness of the MR findings. MVMR analysis indicated that body mass index (BMI), smoking and drinking were not confounding factors. Moreover, bioinformatic analysis identified PARP9, ABCA1, CEACAM1, and DDX60L as promising diagnostic biomarkers for PBC and SLE. These four genes are highly expressed in CD14+ monocytes in PBMCs of SLE patients and potentially associated with innate immune responses and immune activation.

**Conclusion:**

Our study confirmed the bidirectional causal relationship between PBC and SLE and identified PARP9, ABCA1, CEACAM1, and DDX60L genes as the most potentially shared diagnostic genes between the two diseases, providing insights for the exploration of the underlying mechanisms of these disorders.

## Introduction

Systemic lupus erythematosus (SLE) is a complex autoimmune disease that affects multiple organs and systems (1, 2). Genetic factors are widely acknowledged to participate in the development of SLE, even though the specific cause of the disease remains elusive (2–4). Abnormal liver enzymes can be observed in approximately 50% of patients with SLE. Lupus hepatitis and drug-induced hepatitis are common causes of liver enzyme abnormalities, while autoimmune liver diseases such as primary biliary cholangitis (PBC) can also lead to such abnormalities (5–7). PBC is a chronic inflammation of the liver bile ducts that is mediated by autoimmune reactions. Patients with PBC usually present with symptoms such as fatigue, pruritus, and jaundice. Laboratory tests typically show elevated serum alkaline phosphatase, cholestatic liver enzymes, and anti-mitochondrial antibodies. In a few cases, some patients with PBC may develop cirrhosis (8–11). Observational studies have shown that the proportion of patients with PBC also suffering from SLE can reach 3.7% (12–15). Although the prevalence is not very high, there are certain common clinical features between patients with PBC and SLE. One example is the existence of anti-nuclear antibodies (ANA) in their immune profile (13, 14, 16–18). In addition, individuals with SLE-PBC have a higher occurrence of blood, muscle, and pulmonary involvement than those with SLE. Moreover, the combination of PBC also has a negative impact on the survival rate of SLE (19). Thus, it is essential for clinicians to have an exhaustive understanding of this overlap. However, whether PBC and SLE have a causal relationship or their coexistence is coincidental is uncertain. Confounding factors and reverse causation influence the results of observational studies (20, 21). Therefore, a more robust study design is needed to assess the causal relationship between PBC and SLE.

Utilizing genetic variations as instrumental variables (IVs), Mendelian randomization (MR) provides insight into the causal connection between exposure factors and clinical outcomes (22, 23). This approach is valuable in assessing and identifying potential causal associations, especially when confounding factors can bias causal relationships in observational studies, and randomized controlled trials are challenging to implement (24, 25). Through MR analysis, this study seeks to establish the bidirectional causal relationship between PBC and SLE. There is no substantial evidence to establish the mechanistic link between the two diseases. Therefore, this study also utilizes transcriptomic data from PBC and SLE to explore potential interacting genes and shared diagnostic genes between the two diseases to gain insight into the underlying pathological processes that may connect them.

## Materials and methods

### Bidirectional Mendelian randomization

#### Study design and data sources

**Figure 1:**
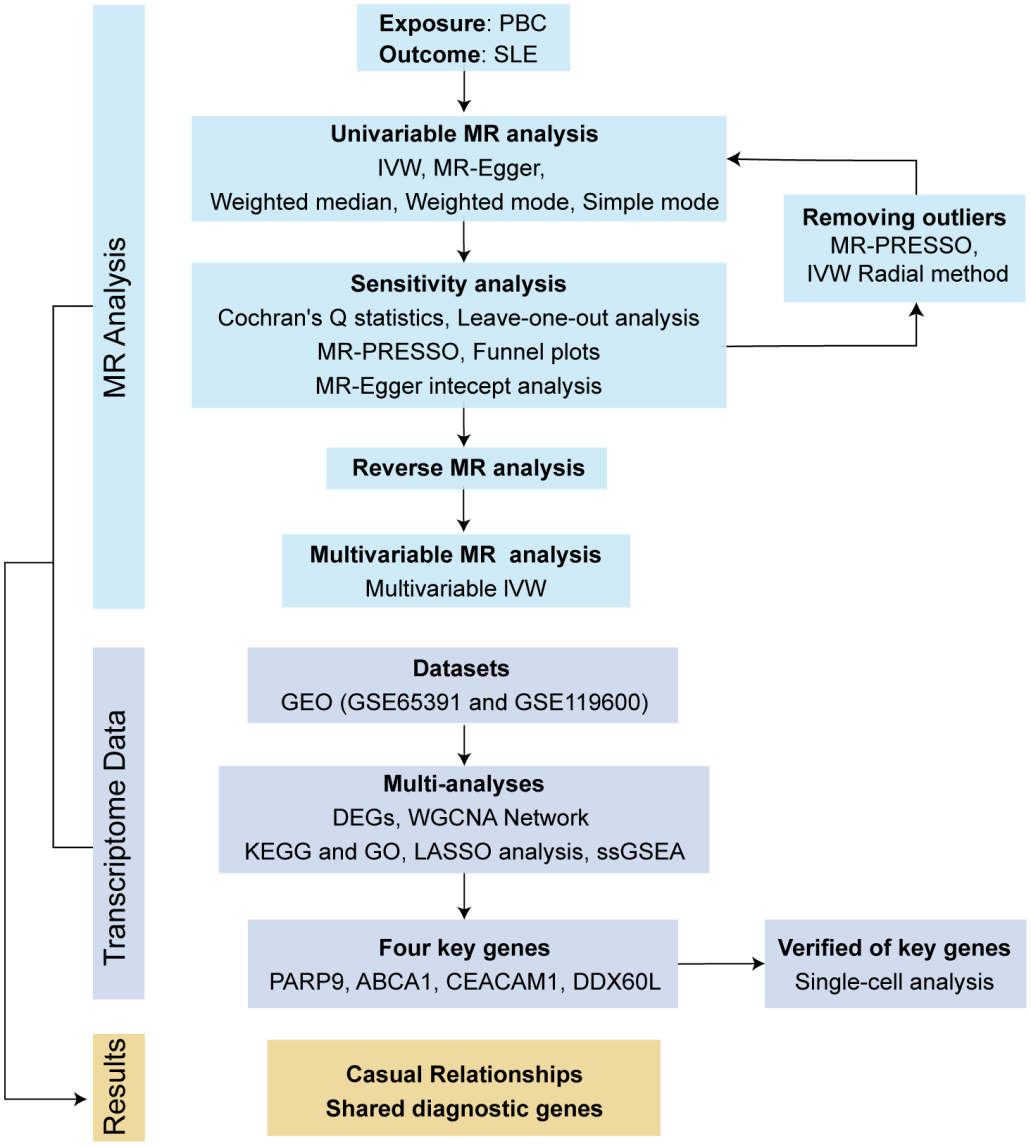
Schematic diagram of the study design.

This investigation employed bidirectional MR analysis to examine the causal connection between PBC and SLE. Genetic association estimates for PBC were obtained from a recent genome-wide association study (GWAS) involving 2764 European PBC patients and 10475 controls (http://gwas.mrcieu.ac.uk/) (26). To increase the reliability of the results, we also used the SNPs of 8021 European PBC patients extracted from a GWAS meta-analysis for validation (27). SLE summary statistics were collected from a large GWAS of the European population comprising 5,201 cases and 9,066 controls (http://gwas.mrcieu.ac.uk/) (28). In addition, a multivariate MR (MVMR) analysis was employed to exclude the influence of confounders, including body mass index (BMI), smoking and alcohol consumption. The dataset for BMI (ukb-b-19953) was obtained from the MRC-IEU consortium, smoking (ieu-b-25) and alcohol consumption (ieu-b-73) were obtained from the GSCAN consortium (https://gwas.mrcieu.ac.uk/datasets/). The study flow diagram is outlined in **Figure 1**.

#### SNPs selection

During the initial selection phase, we employed strict criteria to choose preliminary candidate single nucleotide polymorphisms (SNPs) for our study. We focused on autosomal biallelic SNPs with a P-value below 5×10^−8^. To ensure the independence of these genetic variants, we conducted a clumping analysis, specifically examining linkage disequilibrium with an R^2^ value below 0.001 within a 10000 kb window. This analysis helped us identify SNPs that were not closely linked to each other. To further validate our chosen SNPs, we manually searched for them in the PhenoScanner V2, a comprehensive database (http://www.phenoscanner.medschl.cam.ac.uk/). This step aimed to confirm that none of the selected SNPs had potential confounding effects on each other. By ensuring their independence and lack of confounding, we increased the reliability of our findings. Additionally, we sought to ensure a robust association between IVs and exposure factors. To achieve this, we excluded IVs with F values below 10, as calculated using the formula (R2/(1-R2) *(N-2)] (29). This step allowed us to prioritize IVs that exhibited strong associations with the relevant exposure factors, thereby enhancing the validity and accuracy of our study.

#### Statistical analysis

In this study, we employed a diverse set of five methods to assess the bidirectional causal effects between PBC and SLE. These methods included inverse-variance weighted (IVW), weighted median, simple mode, weighted mode, and MR-Egger. The IVW method served as our primary approach, as it offers reliable estimates in the absence of pleiotropy and heterogeneity. For MVMR analysis, the multivariate IVW method was employed. To address potential issues like pleiotropy, we conducted an MR-Egger regression analysis. We also evaluated heterogeneity using Cochran’s Q statistics. Additionally, we performed sensitivity analyses using leave-one-out techniques and funnel plots. Given the potential presence of horizontal pleiotropy, we utilized the MR-PRESSO method to filter out SNPs that could introduce bias and yield more reliable causal inference results. Moreover, we employed the IVW Radial method, which enabled us to generate radial plots and identify any outlier SNPs.

To determine the significance of the causal relationship, we considered multiple factors. A causal relationship was deemed significant if the P value obtained from the IVW method was less than 0.05. Furthermore, the P value from the MR-Egger intercept test needed to be greater than 0.05, indicating the absence of directional pleiotropy. Additionally, there should be consistency in the direction of estimates among the IVW, MR-Egger, and weighted median methods. The “TwoSampleMR,” “MRPRESSO,” “MVMR,” and “MendelianRandomization,” package for R version 4.3.0 was used to perform all statistical analyses.

### Transcriptomic analyses

#### Data collection and download

We utilized gene expression data from two datasets to investigate the association between PBC and SLE. The first dataset, GSE119600, sourced from the GEO database (https://www.ncbi.nlm.nih.gov/geo/), comprised microarray-based measurements using RNA from whole blood samples. It included 370 samples, of which we selected 90 individuals diagnosed with PBC and 47 healthy controls for data analysis. The second dataset, GSE65391, also obtained from the GEO database, consisted of 996 samples, including 924 SLE cases and 72 healthy controls. Those datasets provided valuable insights into gene expression patterns related to PBC and SLE.

#### Differential expression analysis

In the initial analysis, we utilized R (4.3.0) software to process and normalize the expression matrix. By employing the “DEseq2” R package, we identified differentially expressed genes (DEGs) from GSE119600 and GSE65391 datasets, considering an adjusted P value < 0.05. To visually represent the differential gene patterns, we utilized R software to create a heatmap to cluster the DEGs. The Gene Ontology (GO) analysis was also employed using the “ClusterProfiler” R package to delve deeper into the biological mechanisms of DEGs.

#### WGCNA network building and module identification

Utilizing WGCNA, a powerful bioinformatic analysis approach, assists in unraveling the gene correlation patterns across various samples. To construct the co-expression network, we utilized the WGCNA R package and considered genes with an adjusted P value < 0.05. The procedure began with hierarchical clustering to identify outliers utilizing the “Hculst” function. Next, we achieved a scale-free network by selecting the optimal soft thresholding power β through the “pick Soft Threshold” function. The matrix portraying the similarities in gene expression was subsequently transformed into an adjacency matrix by utilizing the “adjacency” function, incorporating the selected soft-thresholding parameter β. To enhance data quality and reduce spurious associations, we converted the adjacency matrix into a topological overlap matrix (TOM). Subsequently, we identified modules using hierarchical clustering and the dynamic tree-cut method. In order to determine the correlation between modules and patient clinical characteristics, Pearson correlation analysis was carried out, with results deemed statistically significant at P < 0.05.

#### Detection of shared genes and pathway enrichment

Through the utilization of Venn diagrams, we conducted an integrated assessment of the genes derived from both WGCNA and DEGs. The resultant common genes were deemed central shared genes and retained for further investigation into functional enrichment. Using R packages like “clusterProfiler,” “enrich plot,” and “ggplot2”, we conducted GO analysis and Kyoto Encyclopedia of Genes and Genomes (KEGG) pathway enrichment analyses. The resulting bar chart displays the top 10 pathways, with p-values below 0.01, highlighting their significance.

#### Identification of shared diagnostic genes using LASSO regression analysis

Utilizing the “glmnet” R package, we employed LASSO regression analysis, a popular technique that employs an ℓ1 penalty to achieve a sparse solution. This analysis aimed to identify the most influential predictors of SLE and PBC among the DEGs and the intersecting genes identified by WGCNA. LASSO regression allowed us to pinpoint the critical factors associated with these diseases, aiding in understanding their underlying mechanisms.

#### Evaluation of shared diagnostic gene expression levels

Using the “GSVA” R package, we performed single-sample gene set enrichment analysis (ssGSEA) to score the shared core genes separately in patient and normal samples. This approach allows us to quantify the degree to which a group of genes shows coherent overexpression or underexpression in an individual sample. To further examine the expression levels of these crucial genes, we employed boxplots from the “gglplot2” package in R, considering a significance threshold of P < 0.05. This visualization technique concisely represents how pivotal genes are expressed across diverse conditions, enhancing our understanding of their functional roles.

#### Single-cell data processing and clustering

The single-cell sequencing analysis started with downloading the raw data from the GSE174188 dataset on GEO, which was then processed using the Seurat software package (version 5.0.0). As per the established protocols, quality control of the scRNA-seq data was stringently performed. Subsequently, we focused on each sample’s top 2000 highly variable genes (HVGs), identified based on variance stabilizing transformation (vst). These HVGs were then normalized for further analysis. We scaled these genes using the ScaleData function, and dimensionality reduction was performed using the RunPCA function, where we specifically chose 30 dimensions (dim = 30). Following this, cell clustering was conducted using the FindNeighbors and FindClusters functions, identifying 17 distinct cell clusters. These clusters were visualized using the RunUMAP function, allowing for a clear representation of the cellular heterogeneity and the distinct cell populations within the dataset.

## Result

### Causal effects of PBC on SLE and sensitivity analyses

To evaluate the causal effects of PBC on SLE, we analyzed 1,124,241 PBC-related SNPs from the GWAS study involving 2,764 European PBC patients and 10475 controls. To remove the influence of confounders, we excluded SNPs strongly correlated with SLE (rs10488631, rs2304256, rs9303277) using the PhenoScanner V2 website, resulting in a preliminary selection of 21 SNPs as IVs for PBC (**Supplementary Table S1**).

Our primary analyses observed a significant causal association between PBC and an SLE (IVW, OR: 1.319, 95% CI: 1.168 - 1.489, P < 0.001). The other four MR methods revealed similar results (**Supplementary Table S2**). MR-Egger regression analyses showed no pleiotropy (Intercept, 0.034, P = 0.603). However, Cochran’s Q test showed the exist of heterogeneity (IVW, Q =131.319, P < 0.001; MR-Egger, Q = 129.418, P < 0.001) (**Supplementary Table S3**). To address this concern, we utilized the MR-PRESSO (rs35464393, rs3771317) and the IVW Radial method (rs11065987, rs12924729, rs3745516) to identify outlier SNPs (**Supplementary Figure S1**).

**Figure 2:**
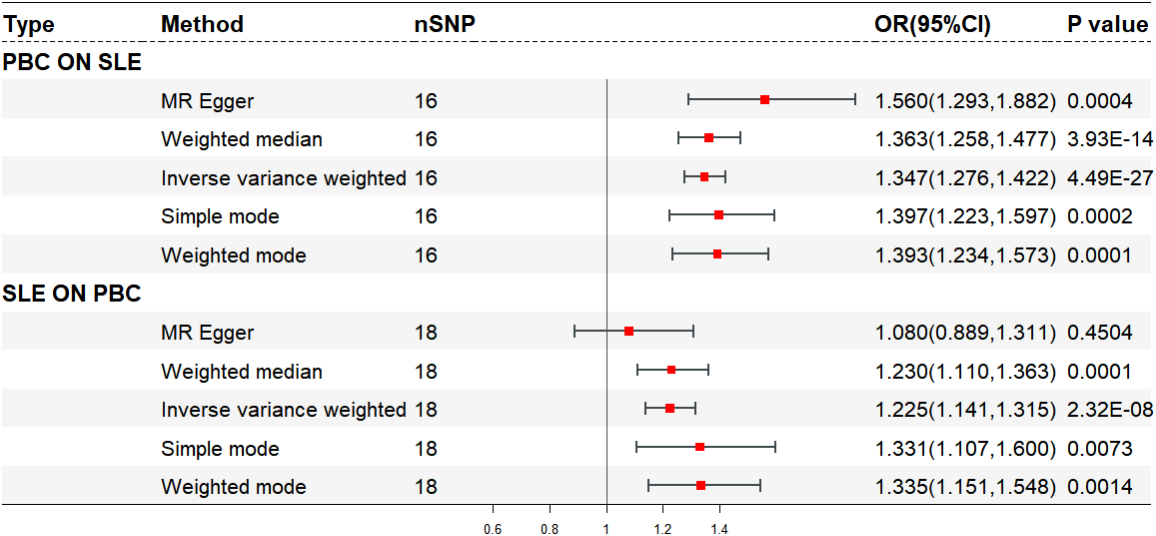
Forest plot of bidirectional mendelian randomization.

**Figure 3:**
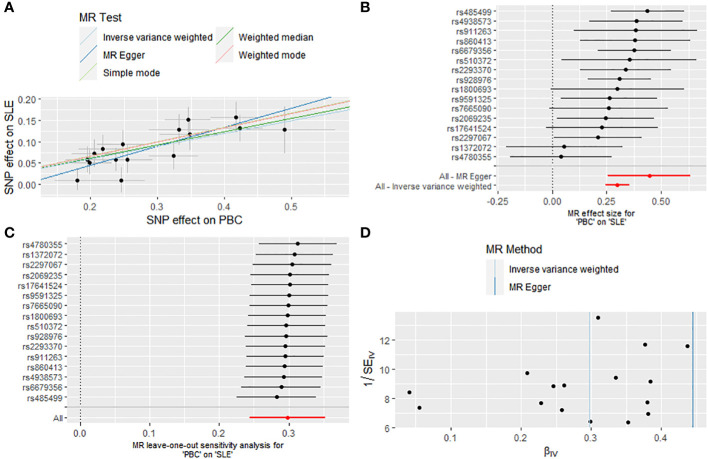
Causal effect of PBC on the risk of SLE. **(A)** Scatter plot. **(B)** Forest plot. **(C)** Leave-one-out test. **(D)** Funnel plot.

After excluding five outlier SNPs, the final analysis still demonstrated a significant and consistent causal association between PBC and an increased risk of SLE. The OR estimates obtained from five MR methods were as follows: 1.347 (95% CI: 1.276 - 1.422) for the IVW method, 1.560 (95% CI: 1.293 - 1.882) for MR Egger, 1.363 (95% CI: 1.258 - 1.477) for weighted median, 1.393 (95% CI: 1.234 - 1.573) for weighted mode, and 1.397 (95% CI: 1.223 - 1.597) for simple mode, all indicating statistically significant associations between PBC and SLE (P < 0.001 across five MR methods) (**Figures 2**, **3A, B**; **Supplementary Table S2**). Moreover, no evidence of heterogeneity (IVW, Q = 14.418, P = 0.494; MR-Egger, Q = 11.865, P = 0.617) or horizontal pleiotropy (Intercept, -0.043, P = 0.132) was observed (**Supplementary Table S3**). A leave-one-out analysis revealed that no single SNP significantly influenced the causal relationship between PBC and SLE (**Figure 3C**). Moreover, a relatively symmetrical distribution of variant effects for SLE was observed in the Funnel plot (**Figure 3D**).

### Causal effects of SLE on PBC and sensitivity analyses

In reverse analysis, 27 SNPs were screened as IVs for SLE to evaluate causality with PBC (**Supplementary Table S4**). Initial analysis indicated a significant causal relationship between SLE and PBC (IVW, OR: 1.255, 95% CI: 1.116 - 1.411, P < 0.001), details are shown in **Supplementary Table S5**. Sensitivity analyses revealed no evidence of pleiotropy (Intercept, 0.072, P = 0.098). While the Cochran’s Q test identified potential heterogeneity in the initial analysis (IVW, Q = 168.677, P < 0.001; MR-Egger, Q = 150.810, P < 0.001) (**Supplementary Table S6**). Therefore, we employed the MR-PRESSO (rs35000415, rs35251378, rs389884, rs597808) and the IVW Radial method (rs2573219, rs353608, rs4274624, rs6671847, rs9852014) to identify outlier SNPs (**Supplementary Figure S2**).

**Figure 4:**
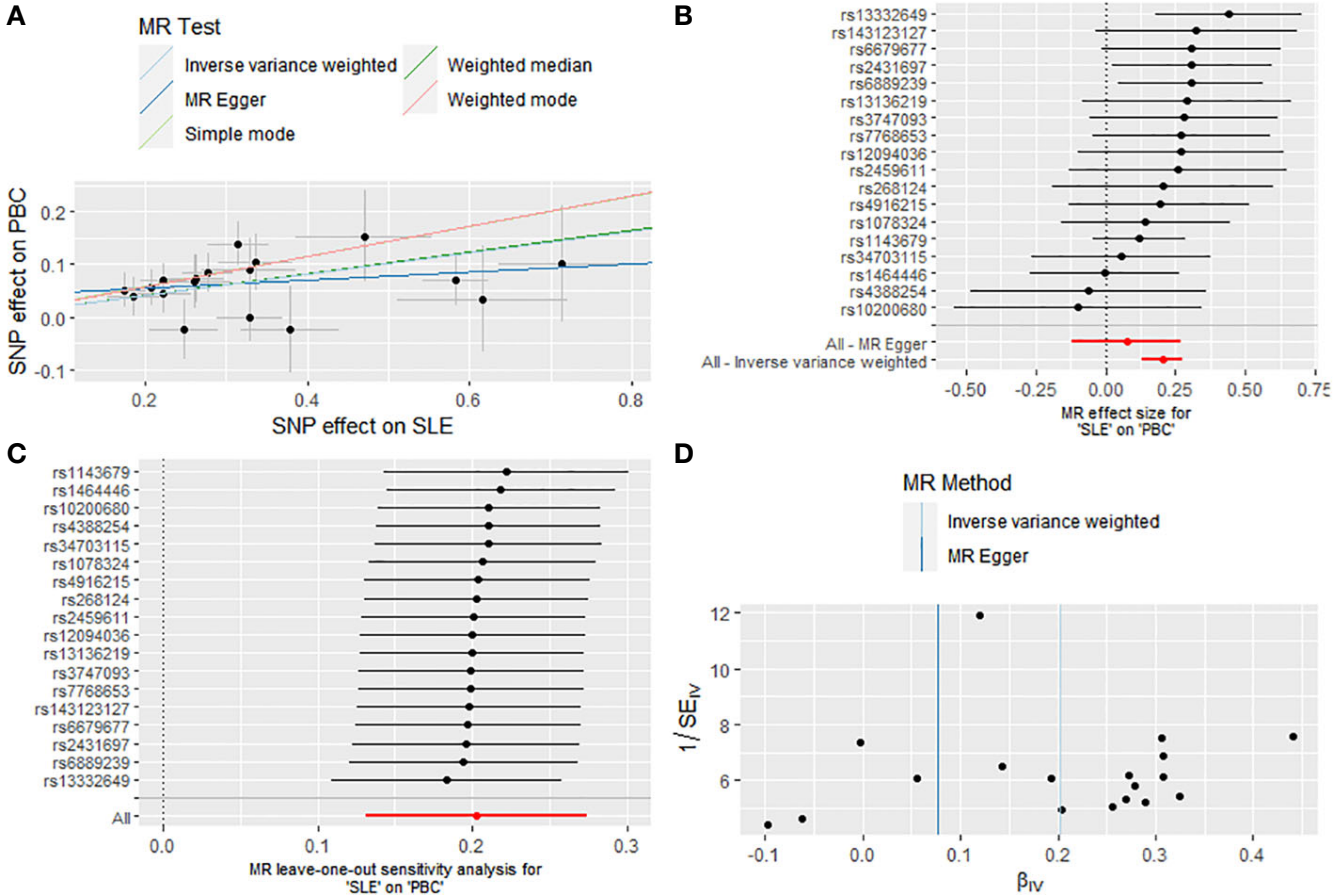
Causal effect of SLE on the risk of PBC. **(A)** Scatter plot. **(B)** Forest plot. **(C)** Leave-one-out test. **(D)** Funnel plot.

The final analysis confirmed the continued presence of a causal connection between SLE and the increased risk of PBC (OR: 1.225, 95% CI: 1.141 - 1.315, P < 0.001 for IVW; OR: 1.230, 95% CI: 1.110 - 1.363, P < 0.001 for weighted median; OR: 1.335, 95% CI: 1.151 - 1.548, P = 0.001 for weighted mode; OR: 1.331, 95% CI: 1.107 - 1.600, P = 0.007 for simple mode; OR: 1.080, 95% CI: 0.889 - 1.311, P = 0.450 for MR Egger) (**Figures 2**, **4A, B**; **Supplementary Table S5**), with no evidence of heterogeneity (IVW, Q = 13.503, P = 0.702; MR-Egger, Q = 11.643, P = 0.768) and pleiotropy (Intercept, 0.040, P = 0.191) (**Supplementary Table S6**). Similarly, we utilized a leave-one-out analysis and funnel plots to supplement the reliability of the results (**Figures 4C, D**).

### Validation of bidirectional MR analysis

To verify the robustness of the results of the bidirectional MR analysis described above, we also used the SNPs of 8021 European PBC patients extracted from a GWAS meta-analysis for analysis. The results of the validation cohort were consistent with previous studies and supported a bidirectional causal relationship between PBC and SLE, as shown in **Supplementary Tables S7**-**12**, **Supplementary Figures S3**, **4**.

### Multivariable MR analysis

Within the MVMR, significant direct causal associations were observed between PBC and an increased risk of SLE (OR = 1.34126, 95% CI: 1.23731 - 1.45393, P < 0.001). Conversely, SLE was also directly associated with an increased risk of PBC (OR = 1.19932, 95% CI: 1.12629 - 1.27708, P < 0.001). BMI, smoking and alcohol consumption did not confound the results (**Supplementary Table S13**).

### Identification of differentially expressed genes

**Figure 5:**
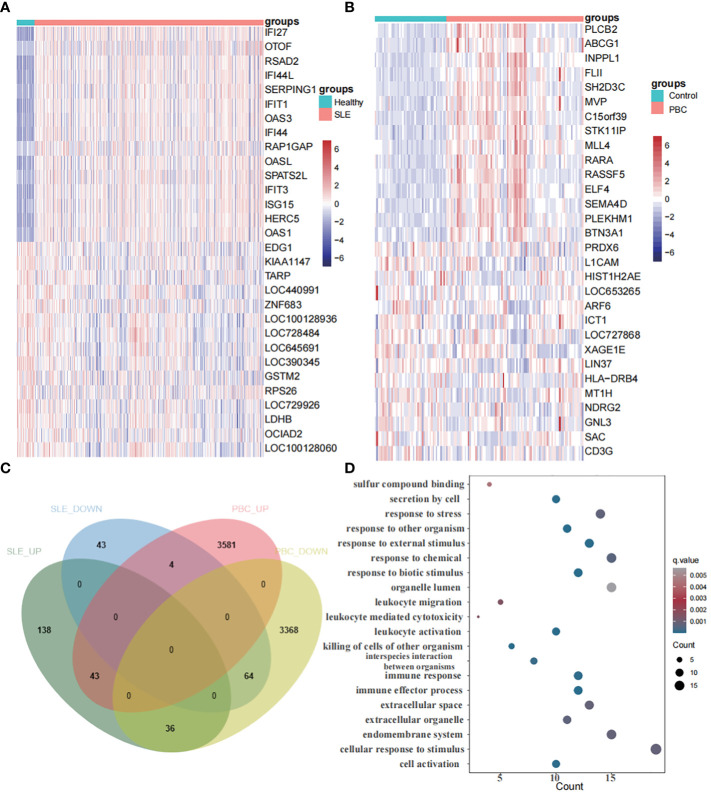
Identification of differentially expressed genes. **(A)** A heatmap of the top 30 DEGs in GSE65391. **(B)** A heatmap of the top 30 DEGs in GSE119600. **(C)** Venn diagram shows that 107 genes overlap in the SLE and PBC. **(D)** Gene ontology enrichment analysis of these 107 genes.

In the SLE dataset (GSE65391), we discovered 328 DEGs, with 217 upregulated and 111 downregulated DEGs. Similarly, in the PBC dataset (GSE119600), we identified 7096 DEGs, comprising 3628 upregulated and 3468 downregulated DEGs. Heatmaps (**Figures 5A, B**) showcased the top 30 DEGs for both diseases. Notably, we found 107 DEGs overlapping between SLE and PBC (**Figure 5C**). Conducting GO enrichment analysis on these 107 genes revealed their significant involvement in immune-related processes, such as immune effector activity, leukocyte activation, cell activation, immune response, and interspecies interaction between organisms (**Figure 5D**).

### Correlation analysis between modules and clinical traits with WGCNA

**Figure 6:**
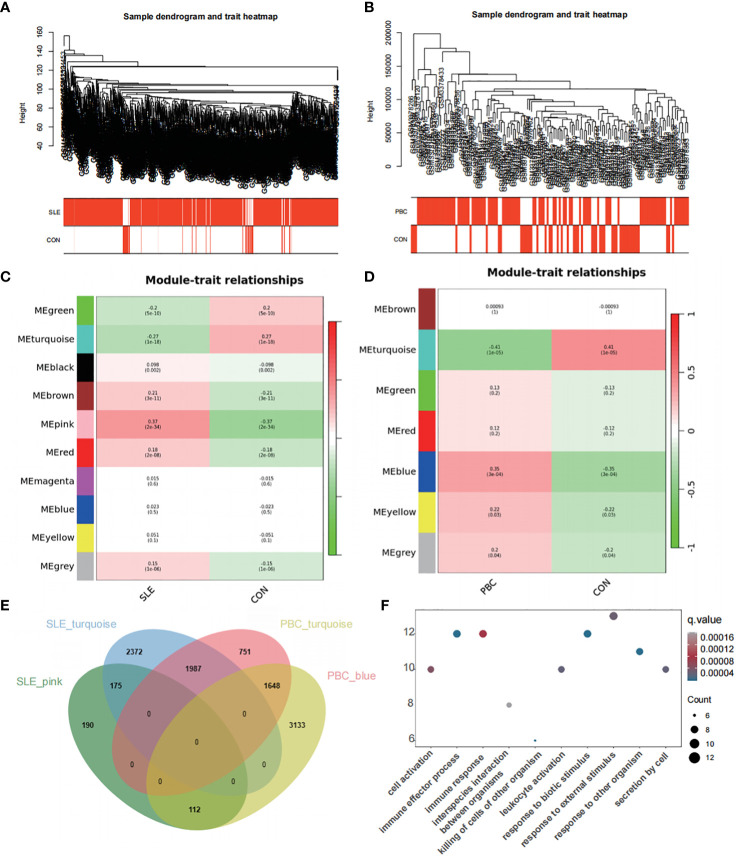
Co-expression network analysis for differentially expressed genes. **(A)** Sample dendrogram and trait heatmap in GSE65391 **(B)** Sample dendrogram and trait heatmap in GSE119600. **(C)** Heatmap of the module-trait relationships in GSE65391. **(D)** Heatmap of the module-trait relationships in GSE119600. **(E)** Venn diagram identifies 112 shared genes by overlapping the hub modules of PBC and SLE. **(F)** Gene ontology enrichment analysis of these 112 genes.

Sample clustering was performed to identify aberrant samples. No abnormal samples were detected in GSE65391, whereas 16 outlier samples were removed from GSE119600 (**Figures 6A, B**). To achieve a scale-free network, we evaluated the scale-free fit index and average connectivity. The soft threshold for GSE65391 was determined as β=8, while for GSE119600, β was set to 12. The co-expression network analysis identified ten modules for SLE samples and seven for PBC samples (**Figures 6C, D**).

To explore disease progression-related genes, we assessed the relationship between modules and clinical phenotypes, enabling us to uncover potential genetic associations. In the GSE65391 dataset for SLE, the pink module exhibited the most potent positive correlation (r = 0.37, P < 0.001), whereas the turquoise module showed the most significant negative correlation (r = -0.27, P < 0.001). Similarly, for PBC in the GSE119600 dataset, the blue module displayed the most potent positive correlation (r = 0.35, P < 0.001), while the turquoise module had the most significant negative correlation (r = -0.41, P < 0.001). By overlapping the hub modules of PBC and SLE using a Venn diagram, we identified 112 shared genes (**Figure 6E**).

GO enrichment analysis of these 112 genes revealed their involvement in pathways such as cell activation, immune effector process, immune response, killing of cells of other organisms, and leukocyte activation (**Figure 6F**), similar to the pathways enriched by the DEGs.

### Identification of potential shared genes

**Figure 7:**
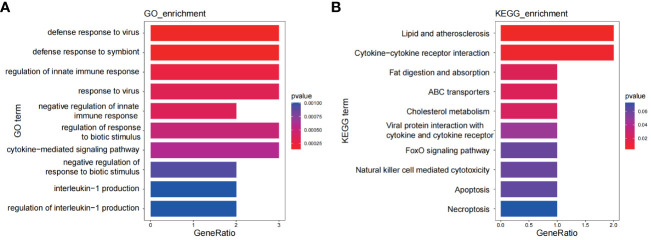
Functional enrichment analyses of the shared genes. **(A)** GO analysis of the shared genes. **(B)** KEGG pathway enrichment analysis of the shared genes.

A set of eight genes (DDX60L, CEACAM1, PARP9, IL1RN, ABCA1, TMEM140, TNFSF10, IFI16) were identified as common genes that intersected between the genes identified through WGCNA and DEGs. These genes hold the potential as crucial links between the two diseases. To explore shared regulatory pathways, we performed GO and KEGG enrichment analyses on these eight genes. GO analysis revealed their association with defense response to virus, response to virus, regulation of innate immune responses, and interleukin-1 production (**Figure 7A**). Additionally, KEGG analysis indicated their involvement in pathways such as lipid and atherosclerosis, cytokine-cytokine receptor interaction, and natural killer cell-mediated cytotoxicity (**Figure 7B**).

### Identification of shared diagnostic genes via LASSO

We then utilized the LASSO regression method to uncover potential shared diagnostic genes. In GSE65391, the LASSO examination pinpointed 4 of the 8 core intersecting genes with the optimal λ = 0.00403 (**Supplementary Figures S5A, B**). Similarly, in GSE119600, the LASSO analysis identified 7 of the 8 core intersecting genes with the most suitable λ = 0.00342 (**Supplementary Figures S5C, D**). As a result, we discovered 4 common genes (PARP9, ABCA1, CEACAM1, DDX60L) as the top shared diagnostic biomarkers for both PBC and SLE (**Supplementary Figure S5E**).

### Evaluation of shared diagnostic gene expression levels

**Figure 8:**
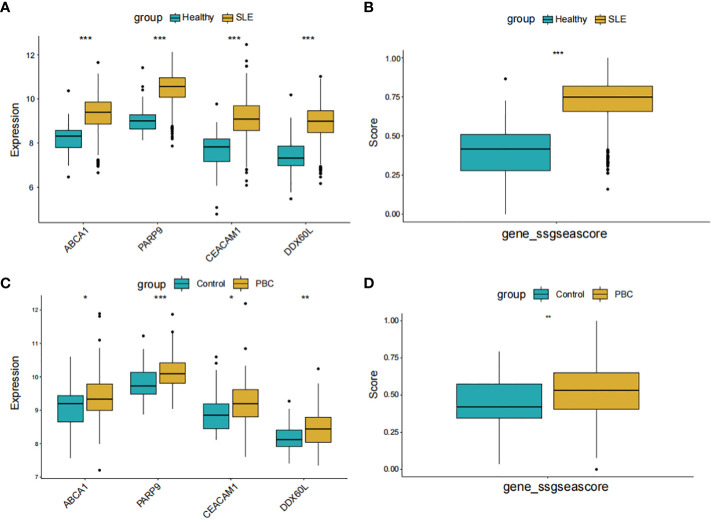
Expression pattern validation and diagnostic value. **(A)** Expression of PARP9, ABCA1, CEACAM1 and DDX60L in GSE65391. **(B)** ssGSEA score of the shared diagnostic genes in GSE65391. **(C)** Expression of PARP9, ABCA1, CEACAM1 and DDX60L in GSE119600. **(D)** ssGSEA score of the shared diagnostic genes in GSE119600. ssGSEA, single-sample gene set enrichment analysis; *P < 0.05; **P < 0.01; ***P < 0.001.

In **Figures 8A, C**, the expression levels of the 4 core genes in the two datasets are displayed. Remarkably, the genes PARP9, ABCA1, CEACAM1, and DDX60L were significantly upregulated in both SLE and PBC (P < 0.05). Furthermore, when scoring these four genes as a gene set for the two datasets, significant differences were observed between the two groups, affirming the potential of these four genes as shared diagnostic biomarkers for both diseases (**Figures 8B, D**).

### Identification of shared diagnostic genes in PBMCs-specific cell populations of SLE patients

**Figure 9:**
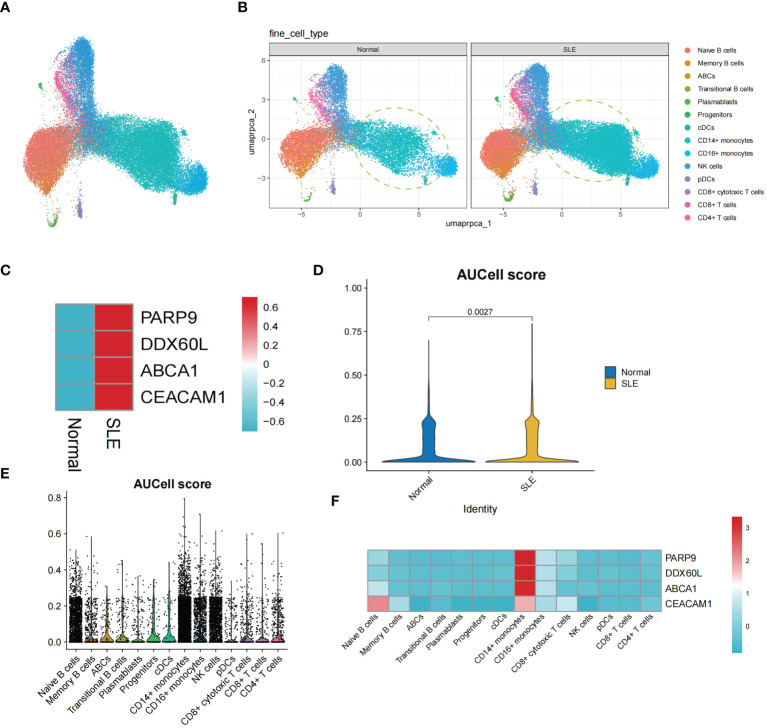
The single-cell transcriptomic analysis of PBMCs in SLE patients. **(A)** UMAP plot illustrating the distribution of 14 identified cell populations within PBMCs. **(B)** Split UMAP plots showcasing the distribution of the 14 cell populations within PBMCs from SLE patients and healthy control groups. **(C)** Heatmap displaying the expression levels of PARP9, ABCA1, CEACAM1, and DDX60L genes in the SLE and healthy PBMC populations. **(D)** Violin plots representing AUCell scoring for PARP9, ABCA1, CEACAM1, and DDX60L across all PBMCs. **(E, F)** AUCell scoring and expression of PARP9, ABCA1, CEACAM1, and DDX60L across the 14 identified cell populations within PBMCs.

The single-cell transcriptomic analysis of PBMCs from SLE patients and healthy individuals revealed 14 distinct cell populations within the PBMCs (**Figure 9A**). Notably, we observed an increased presence of CD14+ monocytes in the PBMCs of SLE patients compared to healthy individuals (**Figure 9B**). Upon conducting an expression analysis of the identified Shared Diagnostic Genes (PARP9, ABCA1, CEACAM1, DDX60L) in the PBMCs of both SLE patients and healthy individuals, it was found that these genes exhibited higher expression levels in the PBMCs of SLE patients (**Figure 9C**). This finding underscores the potential feasibility of these four genes as shared diagnostic biomarkers. Further, after performing aucell scoring using these four genes on the PBMCs, it was evident that their expression was significantly higher in SLE patients compared to healthy individuals (P < 0.05, **Figure 9D**). Most compellingly, a more detailed analysis revealed that the expression of PARP9, ABCA1, CEACAM1, DDX60L was notably elevated in the CD14+ monocyte population compared to other cell groups within the PBMCs (**Figures 9E, F**).

## Discussion

This study conducted a bidirectional MR analysis to investigate the causal relationship between PBC and SLE and integrated transcriptomic data from PBC and SLE to explore shared diagnostic genes between the two diseases. The results revealed a bidirectional causal relationship between PBC and SLE. Specifically, it was observed that PBC increased the risk of SLE (IVW, OR: 1.347, 95% CI: 1.276-1.422, p<0.001), and conversely, SLE could also increase the risk of PBC (IVW, OR: 1.225, 95% CI: 1.141-1.315, p<0.001). In addition, transcriptomic analyses identified PARP9, ABCA1, CEACAM1, and DDX60L as promising diagnostic biomarkers for PBC and SLE. These genes are highly expressed in CD14+ monocytes in PBMCs of SLE patients and may be associated with innate immune responses and immune activation.

The relationship between PBC and SLE is receiving increasing attention, and several retrospective and descriptive studies have summarized the coexistence of PBC and SLE. Overall, the current studies suggest that the prevalence of SLE is higher in patients with PBC than in the control population (12, 14). The two diseases have some common clinical features, and their coexistence may complicate organ involvement and worsen clinical prognosis (16–19). However, the exact causal relationship between the two diseases is uncertain. This study utilized MR analysis to provide evidence for a bidirectional causal relationship between PBC and SLE from a genetic perspective. At the same time, we validated the results of the MR analysis using another set of datasets, further confirming the reliability of the results. A retrospective study has observed a higher prevalence of SLE in patients with PBC than in the control population and a higher prevalence of SLE in relatives of patients with PBC, suggesting a possible genetic link between the two diseases (14). In addition, previous GWAS studies have also identified the IRF5-TNPO3 gene haplotype locus, which is shared by PBC and SLE (30, 31). Recently, a GWAS study has revealed that CD58 is a shared genetic susceptibility locus between SLE and PBC, and rs10924104 is associated with the regulating of CD58 expression and the intensity of autoimmune disease susceptibility (32). These studies provide indirect support for our findings of a genetic association between PBC and SLE.

This study integrated PBC and SLE transcriptome data and identified PARP9, ABCA1, CEACAM1 and DDX60L as potential diagnostic biomarkers. Single-cell analysis detected increased expression of these four genes in PBMCs from SLE patients compared to healthy individuals, mainly in CD14+ monocyte subsets. Notably, CD14+ monocytes were also validated as the dominant cell population in PBMCs from SLE patients, suggesting that CD14+ monocytes in PBMCs may be a key cell population for diagnosing and understanding the common pathogenesis of SLE and PBC. Previous studies have reported that in European and Asian SLE populations, expression levels of common SLE GWAS SNPs are significantly increased in CD14+ monocytes, with significant enrichment for DNase I hypersensitive sites ((DHSs). DHSs are important sites for the regulation of gene transcription and are generally poorly methylated. During the pathogenesis of SLE, CDHs in CD14+ monocytes are mainly related to the regulation of the innate immune responses of and the activation of immune effects (33). Type I interferon (IFN-1) plays a crucial role in the development of SLE (34). Studies have observed that there are highly expressed gene transcripts that induce IFN-1 production in monocytes of SLE patients, and these IFN-1 regulatory genes have generally low methylation levels, including the PARP9 (35, 36). In PBC, it has been reported that the number of CD14+ monocytes is increased in PBC and that this is associated with α-SMA-positive myofibroblasts in liver tissue (37). However, the expression of these four diagnostic genes in PBC CD14+ monocytes cannot be further investigated due to the current lack of single-cell transcriptomic data, and we will continue to investigate this area.

PARP9 is a protein-coding gene primarily involved in DNA damage repair, transcriptional regulation and immune responses, including IFN-mediated antiviral defenses (38). PARP9 has been reported to recognize and bind viral RNA, activate the PI3K/AKT pathway, and significantly contribute to the initiation and amplification of IFN-1 generation (39). IFN-1 is an important mediator in the pathogenesis of SLE, and viral infections are considered to be a causative factor in the pathogenesis (35). Therefore, it cannot be excluded that the role of PARP9 in CD14+ monocytes from SLE patients is related to PARP9-mediated production of type I IFN in the initial immune cells. DDX60L belongs to the DExD/H-box RNA helicase family and is involved in RNA metabolism, innate immune responses, and antiviral defense (40). There are few studies on DDX60L and one study have reported that it may be an interferon-stimulated gene product and may enhance IFN-1 production (41). So far, there has been limited research on the role of PARP9 and DDX60L in PBC. ABCA1 is mainly involved in cholesterol and lipid metabolism (42). but it has been reported that ABCA1 is also implicated in phagocytosing apoptotic cells (43–45). In SLE peripheral blood, monocytes and their differentiated macrophages have high capacity to phagocytose apoptotic cells (46). Whether the increased expression of ABCA1 in CD14+ monocytes of SLE patients is related to this physiological process requires further investigation. Interestingly, the pathogenesis of PBC is associated with phagocytosis by apoptotic cholangiocytes (47). The expression of ABCA1 is significantly increased in liver tissue (48), and whether this is directly related to the phagocytosis of apoptotic cholangiocytes remains unclear. CEACAM1 is a signaling receptor. In monocytes, it regulates monocyte survival and differentiation through the ERK/MEK and PI3K/Akt pathways (49). In addition, CEACAM1 can also promote the initiation of T-cell responses (50). CEACAM1 expression is upregulated in bile duct epithelial cells from PBC patients (51), but the exact mechanism of action needs to be verified.

Nevertheless, it is vital to acknowledge the constraints of our study. Firstly, our study only involved individuals of European descent to better control for confounding introduced by heterogeneity. Thus, the applicability of the findings may have some limitations. Secondly, genetic susceptibility and epigenetic modifications contribute to the development of autoimmune diseases, and our study did not specifically address the role of epigenetics in gene expression. Therefore, our conclusions may require further validation and refinement in future studies. Lastly, although our screening process found some shared diagnostic genes for these two diseases, more mechanistic studies are required to verify these results and prove their biological correlation. In the future, we will simultaneously collect clinical patients for further study.

## Conclusion

Our study confirmed the bidirectional causal relationship between PBC and SLE, and identified PARP9, ABCA1, CEACAM1, and DDX60L genes as the most potential shared diagnostic genes between the two diseases, providing insights for exploring the underlying mechanisms of those diseases.

## Data availability statement

The original contributions presented in the study are included in the article/**Supplementary Material**. Further inquiries can be directed to the corresponding authors.

## Ethics statement

The human participants included in this article were gathered from multiple previous studies. This study utilized comprehensive GWAS datasets rather than individual-level data, making ethical approval irrelevant.

## Author contributions

TT: Writing – original draft. AT: Writing – original draft. LL: Writing – original draft. JY: Writing – original draft. LW: Writing – original draft. LZ: Writing – review & editing. JC: Writing – review & editing.
