# Supplementary material for: Investigating the causal relationship and potential shared diagnostic genes between primary biliary cholangitis and systemic lupus erythematosus using bidirectional Mendelian randomization and transcriptomic analyses
Source: Front Immunol. 2024 Feb 19;15:1270401. doi: 10.3389/fimmu.2024.1270401 (PMC10921416; doi:10.3389/fimmu.2024.1270401)
Supplement: Supplementary file 1 [file DataSheet_1.doc]

**Supplementary Material**

Table S1. Details of SNPs associated with PBC on SLE.

Table S2. The causal association between PBC and SLE using five methods.

Table S3. Sensitivity analysis of MR analyses of PBC on SLE.

Table S4. Details of SNPs associated with SLE on PBC.

Table S5. The causal association between SLE and PBC using five methods.

Table S6. Sensitivity analysis of MR analyses of SLE on PBC.

Table S7. Details of SNPs associated with PBC on SLE (Validation cohort).

Table S8. The causal association between PBC and SLE using five methods (Validation cohort).

Table S9. Sensitivity analysis of MR analyses of PBC on SLE (Validation cohort).

Table S10. Details of SNPs associated with SLE on PBC (Validation cohort).

Table S11. The causal association between SLE and PBC using five methods (Validation cohort).

Table S12. Sensitivity analysis of MR analyses of SLE on PBC (Validation cohort).

Table S13. Multivariable MR analysis for evaluating effects of BMI, smoking, and alcohol consumption on the results.

Figure S1. The radial plot of outliers of PBC on SLE.

Figure S2. The Radial plot of outliers of SLE on PBC.

Figure S3. The radial plot of outliers of PBC on SLE (Validation cohort).

Figure S4. The Radial plot of outliers of SLE on PBC (Validation cohort).

Figure S5. Identification of potential shared diagnostic genes by the LASSO regression model.

Table S1. Details of SNPs associated with PBC on SLE.

| **SNP** | **Position** | **Effect allele** | **Other allele** | **BETA** | **EAF** | **SE** | **P-value** | **F-value** |
| --- | --- | --- | --- | --- | --- | --- | --- | --- |
| rs11065987^#^ | 12:112072424 | G | A | 0.17974 | 0.5805 | 0.03250 | 3.20E-08 | 204.990 |
| rs12924729^#^ | 16:11187783 | A | G | -0.27181 | 0.3429 | 0.03563 | 2.39E-14 | 426.031 |
| rs1372072 | 3:16955259 | A | G | 0.17999 | 0.6332 | 0.03278 | 3.99E-08 | 196.190 |
| rs17641524 | 1:197704717 | T | C | 0.25519 | 0.1829 | 0.03750 | 1.01E-11 | 252.629 |
| rs1800693 | 12:6440009 | C | T | 0.19480 | 0.5746 | 0.03240 | 1.84E-09 | 241.004 |
| rs2069235 | 22:39747780 | A | G | 0.23736 | 0.2992 | 0.03548 | 2.23E-11 | 305.361 |
| rs2293370 | 3:119219934 | A | G | -0.34814 | 0.8360 | 0.04437 | 4.26E-15 | 425.303 |
| rs2297067 | 14:103566785 | T | C | 0.32404 | 0.2376 | 0.03647 | 6.34E-19 | 484.392 |
| rs35464393^*^ | 6:32530198 | C | T | 0.38802 | 0.6551 | 0.05344 | 3.85E-13 | 839.312 |
| rs3745516^#^ | 19:50926742 | G | A | -0.33239 | 0.2296 | 0.03568 | 1.22E-20 | 497.157 |
| rs3771317^*^ | 2:191543962 | C | T | 0.34416 | 0.8559 | 0.04510 | 2.32E-14 | 375.441 |
| rs4780355 | 16:11347858 | C | T | -0.24513 | 0.3340 | 0.03644 | 1.74E-11 | 344.413 |
| rs485499 | 3:159745863 | C | T | -0.34531 | 0.3489 | 0.03451 | 1.43E-23 | 678.266 |
| rs4938573 | 11:118741842 | T | C | 0.33226 | 0.1918 | 0.04476 | 1.15E-13 | 437.544 |
| rs510372 | 11:64115137 | T | C | -0.20567 | 0.3827 | 0.03413 | 1.68E-09 | 259.273 |
| rs6679356 | 1:67820194 | T | C | -0.41700 | 0.1720 | 0.03812 | 7.49E-28 | 623.140 |
| rs7665090 | 4:103551603 | G | A | 0.19894 | 0.5070 | 0.03240 | 8.21E-10 | 256.706 |
| rs860413 | 5:35943042 | C | A | -0.24795 | 0.2873 | 0.03769 | 4.73E-11 | 324.872 |
| rs911263 | 14:68753593 | T | C | 0.21853 | 0.6501 | 0.03655 | 2.25E-09 | 281.334 |
| rs928976 | 6:33049211 | T | C | 0.42291 | 0.6710 | 0.03418 | 3.74E-35 | 962.749 |
| rs9591325 | 13:50811220 | C | T | -0.48988 | 0.0547 | 0.07588 | 1.07E-10 | 320.362 |

Abbreviations: PBC, primary biliary cholangitis; SLE, systemic lupus erythematosus; SNP, single nucleotide polymorphism; SE, standard error. * Outlier SNPs removed by MR-PRESSO; # Outlier SNPs removed by IVW Radial method.

Table S2. The casual association between PBC and SLE using various methods.

| **Exposure** | **Outcome** | **Method** | **Nsnp** | **B** | **Se** | **P-value** | **OR** | **LowerCI** | **UowerCI** |
| --- | --- | --- | --- | --- | --- | --- | --- | --- | --- |
| **The preliminary results of the causal relationship between PBC and SLE** | | | | | | | | | |
| PBC | SLE | MR Egger | 21 | 0.16331 | 0.22398 | 0.475 | 1.17741 | 0.75905 | 1.82635 |
| PBC | SLE | Weighted median | 21 | 0.30805 | 0.03788 | 4.25E-16 | 1.36077 | 1.26339 | 1.46565 |
| PBC | SLE | Inverse variance weighted | 21 | 0.27684 | 0.06193 | 7.82E-06 | 1.31895 | 1.16819 | 1.48918 |
| PBC | SLE | Simple mode | 21 | 0.30210 | 0.06037 | 6.81E-05 | 1.35269 | 1.20174 | 1.52260 |
| PBC | SLE | Weighted mode | 21 | 0.29872 | 0.05456 | 2.33E-05 | 1.34813 | 1.21139 | 1.50030 |
| **The ultimate results of the causal relationship between PBC and SLE** | | | | | | | | | |
| PBC | SLE | MR Egger | 16 | 0.44454 | 0.09571 | 0.0004 | 1.55978 | 1.29298 | 1.88163 |
| PBC | SLE | Weighted median | 16 | 0.30977 | 0.04096 | 3.93E-14 | 1.36311 | 1.25796 | 1.47705 |
| PBC | SLE | Inverse variance weighted | 16 | 0.29814 | 0.02767 | 4.49E-27 | 1.34735 | 1.27623 | 1.42244 |
| PBC | SLE | Simple mode | 16 | 0.33465 | 0.06801 | 0.0002 | 1.39745 | 1.22305 | 1.59673 |
| PBC | SLE | Weighted mode | 16 | 0.33177 | 0.06187 | 7.91E-05 | 1.39343 | 1.23431 | 1.57307 |

Abbreviations: SLE, systemic lupus erythematosus; PBC, primary biliary cholangitis; OR, odds ratio; CI, confidence interval.

Table S3. Sensitivity analysis of MR analyses of PBC on SLE.

| **Outcome** | **Number of IVs** | **Heterogeneity (IVW)** | | **Heterogeneity (MR-Egger)** | | **Pleiotropy test** | | **Outliers** |
| --- | --- | --- | --- | --- | --- | --- | --- | --- |
|  |  | **Q** | **P-value** | **Q** | **P-value** | **Intercept** | **P-value** |  |
| Primary Outcome | 21 | 131.319 | ＜0.001 | 129.418 | ＜0.001 | 0.034 | 0.603 | None |
| Secondary Outcome | 19 | 28.131 | 0.060 | 28.061 | 0.044 | -0.007 | 0.839 | MR-PRESSO  (rs35464393, rs3771317) |
| Final Outcome | 16 | 14.418 | 0.494 | 11.865 | 0.617 | -0.043 | 0.132 | IVW Radial method (rs11065987, rs12924729, rs3745516) |

Abbreviations: IVs: instrumental variables; IVW, inverse-variance weighted; PRESSO, Pleiotropy Residual Sum and Outlier; MR, Mendelian randomization.

Table S4. Details of SNPs associated with SLE on PBC.

| **SNP** | **Position** | **Effect allele** | **Other allele** | **BETA** | **EAF** | **SE** | **P-value** | **F-value** |
| --- | --- | --- | --- | --- | --- | --- | --- | --- |
| rs10200680 | 2:223961877 | T | C | -0.24846 | 0.8559 | 0.04248 | 4.96E-09 | 213.91492 |
| rs1078324 | 5:149202268 | A | C | -0.71335 | 0.0497 | 0.07817 | 7.11E-20 | 652.72512 |
| rs1143679 | 16:31301478 | A | G | 0.58222 | 0.1312 | 0.03999 | 5.03E-48 | 1017.17318 |
| rs12094036 | 1:183561725 | C | T | -0.32850 | 0.0815 | 0.05786 | 1.37E-08 | 226.74926 |
| rs13136219 | 4:102746780 | T | C | -0.17435 | 0.6203 | 0.02779 | 3.50E-10 | 201.34420 |
| rs13332649 | 16:85977502 | G | A | -0.31471 | 0.8022 | 0.03757 | 5.43E-17 | 434.27515 |
| rs143123127 | 17:38010815 | A | G | 0.47000 | 0.0308 | 0.08403 | 2.23E-08 | 185.65352 |
| rs1464446 | 3:146601295 | T | G | -0.32850 | 0.1789 | 0.04015 | 2.79E-16 | 437.92275 |
| rs2431697 | 5:159879978 | C | T | -0.22314 | 0.4314 | 0.02930 | 2.60E-14 | 339.95273 |
| rs2459611 | 2:191939187 | T | C | 0.26137 | 0.1252 | 0.04525 | 7.62E-09 | 210.26258 |
| rs2573219^#^ | 2:233293773 | C | A | 0.58779 | 0.0865 | 0.04293 | 1.13E-42 | 736.34589 |
| rs268124 | 2:65667272 | T | C | 0.18633 | 0.2744 | 0.03237 | 8.60E-09 | 194.49236 |
| rs34703115 | 2:40289046 | C | T | -0.61619 | 0.0328 | 0.10478 | 4.08E-09 | 335.37073 |
| rs35000415^*^ | 7:128594183 | T | C | 0.58779 | 0.8996 | 0.04154 | 1.86E-45 | 834.71443 |
| rs35251378^*^ | 19:10475652 | A | G | -0.23572 | 0.2694 | 0.03243 | 3.61E-13 | 305.19297 |
| rs353608^#^ | 11:35101738 | G | A | 0.18633 | 0.5477 | 0.02802 | 2.93E-11 | 241.15773 |
| rs3747093 | 22:21985094 | A | G | 0.26236 | 0.2018 | 0.03451 | 2.88E-14 | 309.31722 |
| rs389884^*^ | 6:31940897 | G | A | 0.92822 | 0.0736 | 0.04323 | 2.92E-102 | 1479.10099 |
| rs4274624^#^ | 2:191958656 | T | C | -0.55962 | 0.2316 | 0.03268 | 9.73E-66 | 1412.80676 |
| rs4388254 | 5:133431834 | T | C | 0.37844 | 0.9294 | 0.06040 | 3.71E-10 | 263.05961 |
| rs4916215 | 1:173332629 | T | C | 0.22314 | 0.2545 | 0.03397 | 5.07E-11 | 264.43768 |
| rs597808^*^ | 12:112007756 | G | A | -0.16252 | 0.5338 | 0.02947 | 3.51E-08 | 185.06055 |
| rs6671847^#^ | 1:161479745 | A | G | 0.19885 | 0.4871 | 0.02897 | 6.64E-12 | 276.27501 |
| rs6679677 | 1:114303808 | A | C | 0.33647 | 0.0915 | 0.04649 | 4.55E-13 | 263.44703 |
| rs6889239 | 5:150458146 | C | T | 0.27763 | 0.2575 | 0.03174 | 2.19E-18 | 408.05769 |
| rs7768653 | 6:106580629 | T | C | -0.20701 | 0.4016 | 0.02969 | 3.11E-12 | 287.77145 |
| rs9852014^#^ | 3:129084581 | G | A | 0.62058 | 0.9254 | 0.04927 | 2.26E-36 | 718.17811 |

Abbreviations: SLE, systemic lupus erythematosus; PBC, primary biliary cholangitis; SNP, single nucleotide polymorphism; EA, effect allele; OA, other allele; SE, standard error. * Outlier SNPs removed by MR-PRESSO; # Outlier SNPs removed by IVW Radial method.

Table S5. The casual association between SLE and PBC using various methods.

| **Exposure** | **Outcome** | **Method** | **Nsnp** | **B** | **Se** | **P-value** | **OR** | **LowerCI** | **UowerCI** |
| --- | --- | --- | --- | --- | --- | --- | --- | --- | --- |
| **The preliminary results of the causal relationship between SLE and PBC** | | | | | | | | | |
| SLE | PBC | MR Egger | 27 | 0.04959 | 0.11822 | 0.678 | 1.05084 | 0.83350 | 1.32486 |
| SLE | PBC | Weighted median | 27 | 0.13587 | 0.05004 | 0.007 | 1.14554 | 1.03852 | 1.26359 |
| SLE | PBC | Inverse variance weighted | 27 | 0.22701 | 0.06001 | 0.0001 | 1.25484 | 1.11558 | 1.41147 |
| SLE | PBC | Simple mode | 27 | 0.26953 | 0.11845 | 0.031 | 1.30935 | 1.03807 | 1.65153 |
| SLE | PBC | Weighted mode | 27 | -0.05339 | 0.04939 | 0.290 | 0.94801 | 0.86054 | 1.04437 |
| **The ultimate results of the causal relationship between SLE and PBC** | | | | | | | | | |
| SLE | PBC | MR Egger | 18 | 0.07671 | 0.09915 | 0.450 | 1.07973 | 0.88904 | 1.31133 |
| SLE | PBC | Weighted median | 18 | 0.20738 | 0.05237 | 7.49E-05 | 1.23046 | 1.11042 | 1.36347 |
| SLE | PBC | Inverse variance weighted | 18 | 0.20259 | 0.03627 | 2.32E-08 | 1.22457 | 1.14054 | 1.31479 |
| SLE | PBC | Simple mode | 18 | 0.28581 | 0.09389 | 0.007 | 1.33084 | 1.10714 | 1.59974 |
| SLE | PBC | Weighted mode | 18 | 0.28898 | 0.07558 | 0.001 | 1.33507 | 1.15124 | 1.54825 |

Abbreviations: SLE, systemic lupus erythematosus; PBC, primary biliary cholangitis; OR, odds ratio; CI, confidence interval.

Table S6. Sensitivity analysis of MR analyses of SLE on PBC.

| **Outcome** | **Number of IVs** | **Heterogeneity (IVW)** | | **Heterogeneity (MR-Egger)** | | **Pleiotropy test** | | **Outliers** |
| --- | --- | --- | --- | --- | --- | --- | --- | --- |
|  |  | **Q** | **P-value** | **Q** | **P-value** | **Intercept** | **P-value** |  |
| Primary Outcome | 27 | 168.677 | ＜0.001 | 150.810 | ＜0.001 | 0.072 | 0.098 | None |
| Secondary Outcome | 23 | 44.889 | 0.003 | 43.294 | 0.003 | 0.027 | 0.389 | MR-PRESSO  (rs35000415, rs35251378, rs389884, rs597808) |
| Final Outcome | 18 | 13.503 | 0.702 | 11.643 | 0.768 | 0.040 | 0.191 | IVW Radial method (rs2573219, rs353608, rs4274624, rs6671847, rs9852014) |

Abbreviations: IVs: instrumental variables; IVW, inverse-variance weighted; PRESSO, Pleiotropy Residual Sum and Outlier; MR, Mendelian randomization.

Table S7. Details of SNPs associated with PBC on SLE (Validation cohort).

| **SNP** | **Position** | **Effect allele** | **Other allele** | **BETA** | **EAF** | **SE** | **P-value** | **F-value** |
| --- | --- | --- | --- | --- | --- | --- | --- | --- |
| rs11117432^#^ | 16:86019271 | A | G | -0.27323 | 0.2187 | 0.02686 | 2.82E-24 | 641.62082 |
| rs1119132^#^ | 16:27403469 | G | A | 0.20264 | 0.8887 | 0.03281 | 6.58E-10 | 200.71548 |
| rs12531711^*^ | 7:128617466 | G | A | 0.42020 | 0.1022 | 0.03135 | 8.57E-41 | 820.70206 |
| rs137687 | 22:39740078 | A | G | -0.21776 | 0.4374 | 0.02187 | 2.37E-23 | 585.65328 |
| rs1800693 | 12:6440009 | C | T | 0.17976 | 0.5746 | 0.02170 | 1.19E-16 | 393.36668 |
| rs1808094^#^ | 18:67526026 | C | T | -0.12774 | 0.5249 | 0.02149 | 2.79E-09 | 201.09229 |
| rs2293370 | 3:119219934 | A | G | -0.29901 | 0.8360 | 0.02894 | 5.54E-25 | 615.93706 |
| rs2327832^#^ | 6:137973068 | G | A | 0.16116 | 0.8320 | 0.02542 | 2.31E-10 | 179.24388 |
| rs2546890 | 5:158759900 | G | A | -0.14423 | 0.4911 | 0.02161 | 2.50E-11 | 257.51889 |
| rs3131789^*^ | 6:31020821 | G | A | 0.18017 | 0.5775 | 0.02191 | 2.00E-16 | 394.48974 |
| rs34655300^#^ | 2:25514333 | T | C | 0.13667 | 0.5507 | 0.02200 | 5.23E-10 | 228.64533 |
| rs35127065^*^ | 6:29303901 | T | C | 0.15907 | 0.8280 | 0.02893 | 3.82E-08 | 177.90907 |
| rs3745516 | 19:50926742 | G | A | -0.27432 | 0.2296 | 0.02397 | 2.65E-30 | 670.27957 |
| rs3771317^#^ | 2:191543962 | C | T | 0.28950 | 0.8559 | 0.02977 | 2.40E-22 | 517.37293 |
| rs3784099 | 14:68749927 | A | G | -0.20302 | 0.3439 | 0.02437 | 8.31E-17 | 464.46627 |
| rs4780355^#^ | 16:11347858 | C | T | -0.19955 | 0.3340 | 0.02402 | 1.01E-16 | 442.00703 |
| rs4936443 | 11:118740864 | T | C | 0.36710 | 0.1899 | 0.02974 | 5.39E-35 | 1060.10548 |
| rs589446 | 3:159733527 | T | G | -0.35309 | 0.6501 | 0.02244 | 1.96E-55 | 1473.66691 |
| rs59643720 | 14:103564807 | C | A | 0.31641 | 0.7644 | 0.02445 | 2.73E-38 | 916.83240 |
| rs60600003 | 7:37382465 | G | T | 0.25326 | 0.0974 | 0.03501 | 4.70E-13 | 279.55346 |
| rs6550965 | 3:25383587 | A | C | 0.16309 | 0.4205 | 0.02154 | 3.65E-14 | 321.88453 |
| rs6679356 | 1:67820194 | T | C | -0.43936 | 0.1720 | 0.02614 | 6.61E-63 | 1425.94352 |
| rs7130339^*^ | 11:646232 | A | G | 0.12165 | 0.4841 | 0.02226 | 4.66E-08 | 182.49414 |
| rs72699866^*^ | 14:93116351 | A | G | -0.19516 | 0.1859 | 0.02934 | 2.89E-11 | 285.82712 |
| rs7674640 | 4:103540780 | T | C | 0.21642 | 0.5080 | 0.02209 | 1.56E-22 | 587.56316 |
| rs7805218 | 7:20378801 | A | G | 0.12854 | 0.3539 | 0.02343 | 4.12E-08 | 186.58430 |
| rs79577483 | 16:68036939 | G | A | 0.21174 | 0.8588 | 0.03125 | 1.23E-11 | 269.42069 |
| rs8067378 | 17:38051348 | G | A | 0.25963 | 0.4851 | 0.02153 | 1.75E-33 | 854.06018 |
| rs859767 | 2:135341200 | G | A | -0.13931 | 0.5636 | 0.02306 | 1.54E-09 | 236.20695 |
| rs867436^*^ | 1:2523723 | T | C | 0.13417 | 0.6660 | 0.02262 | 2.99E-09 | 197.85269 |
| rs928976 | 6:33049211 | T | C | 0.41055 | 0.6710 | 0.02247 | 1.50E-74 | 1970.47721 |
| rs9533122^*^ | 13:43055002 | G | A | 0.15513 | 0.4632 | 0.02153 | 5.83E-13 | 296.83215 |
| rs9591325 | 13:50811220 | C | T | -0.45188 | 0.0547 | 0.05017 | 2.14E-19 | 528.71080 |

Abbreviations: SLE, systemic lupus erythematosus; PBC, primary biliary cholangitis; SNP, single nucleotide polymorphism; EA, effect allele; OA, other allele; SE, standard error. * Outlier SNPs removed by MR-PRESSO; # Outlier SNPs removed by IVW Radial method.

Table S8. The casual association between SLE and PBC using various methods (Validation cohort).

| **Exposure** | **Outcome** | **Method** | **Nsnp** | **B** | **Se** | **P-value** | **OR** | **LowerCI** | **UowerCI** |
| --- | --- | --- | --- | --- | --- | --- | --- | --- | --- |
| **The preliminary results of the causal relationship between PBC and SLE** | | | | | | | | | |
| PBC | SLE | MR Egger | 33 | 0.57688 | 0.17584 | 2.56E-03 | 1.78048 | 1.26143 | 2.51310 |
| PBC | SLE | Weighted median | 33 | 0.33795 | 0.03889 | 3.63E-18 | 1.40208 | 1.29917 | 1.51313 |
| PBC | SLE | Inverse variance weighted | 33 | 0.40870 | 0.06597 | 5.82E-10 | 1.50486 | 1.32233 | 1.71259 |
| PBC | SLE | Simple mode | 33 | 0.33706 | 0.05407 | 5.52E-07 | 1.40082 | 1.25996 | 1.55742 |
| PBC | SLE | Weighted mode | 33 | 0.34231 | 0.03949 | 6.63E-10 | 1.40820 | 1.30331 | 1.52153 |
| **The ultimate results of the causal relationship between PBC and SLE** | | | | | | | | | |
| PBC | SLE | MR Egger | 19 | 0.29148 | 0.07962 | 1.94E-03 | 1.33841 | 1.14502 | 1.56446 |
| PBC | SLE | Weighted median | 19 | 0.34440 | 0.03993 | 6.39E-18 | 1.41115 | 1.30492 | 1.52602 |
| PBC | SLE | Inverse variance weighted | 19 | 0.35015 | 0.02799 | 6.43E-36 | 1.41928 | 1.34352 | 1.49930 |
| PBC | SLE | Simple mode | 19 | 0.33314 | 0.06109 | 3.52E-05 | 1.39535 | 1.23790 | 1.57283 |
| PBC | SLE | Weighted mode | 19 | 0.34348 | 0.05397 | 5.39E-06 | 1.40984 | 1.26833 | 1.56714 |

Abbreviations: SLE, systemic lupus erythematosus; PBC, primary biliary cholangitis; OR, odds ratio; CI, confidence interval.

Table S9. Sensitivity analysis of MR analyses of PBC on SLE (Validation cohort).

| **Outcome** | **Number of IVs** | **Heterogeneity (IVW)** | | **Heterogeneity (MR-Egger)** | | **Pleiotropy test** | | **Outliers** |
| --- | --- | --- | --- | --- | --- | --- | --- | --- |
|  |  | **Q** | **P-value** | **Q** | **P-value** | **Intercept** | **P-value** |  |
| Primary Outcome | 33 | 249.635 | ＜0.001 | 241.349 | ＜0.001 | -0.043 | 0.310 | None |
| Secondary Outcome | 26 | 64.714 | ＜0.001 | 64.260 | ＜0.001 | 0.012 | 0.684 | MR-PRESSO  (rs12531711, rs3131789, rs35127065, rs7130339, rs72699866, rs867436, rs9533122) |
| Final Outcome | 19 | 13.525 | 0.759 | 12.906 | 0.742 | 0.017 | 0.442 | IVW Radial method (rs11117432, rs1119132, rs1808094, rs2327832, rs34655300, rs3771317, rs4780355) |

Abbreviations: IVs: instrumental variables; IVW, inverse-variance weighted; PRESSO, Pleiotropy Residual Sum and Outlier; MR, Mendelian randomization.

Table S10. Details of SNPs associated with SLE on PBC (Validation cohort).

| **SNP** | **Position** | **Effect allele** | **Other allele** | **BETA** | **EAF** | **SE** | **P-value** | **F-value** |
| --- | --- | --- | --- | --- | --- | --- | --- | --- |
| rs1078324^*^ | 5:149202268 | A | C | -0.71335 | 0.0497 | 0.07817 | 7.11E-20 | 652.72512 |
| rs1143679 | 16:31283996 | A | G | 0.58222 | 0.1312 | 0.03999 | 5.03E-48 | 1017.17317 |
| rs12524498 | 6:31444187 | T | G | -0.67335 | 0.9891 | 0.12079 | 2.48E-08 | 138.09473 |
| rs13136219 | 4:102743687 | T | C | -0.17435 | 0.6203 | 0.02779 | 3.50E-10 | 201.34420 |
| rs13332649 | 16:85974961 | G | A | -0.31471 | 0.8022 | 0.03757 | 5.43E-17 | 434.27515 |
| rs143123127 | 17:38007190 | A | G | 0.47000 | 0.0308 | 0.08403 | 2.23E-08 | 185.65352 |
| rs1464446^*^ | 3:146601295 | T | G | -0.32850 | 0.1789 | 0.04015 | 2.79E-16 | 437.92275 |
| rs2431697 | 5:159879978 | C | T | -0.22314 | 0.4314 | 0.02930 | 2.60E-14 | 339.95273 |
| rs2573219^*^ | 2:233288667 | C | A | 0.58779 | 0.0865 | 0.04293 | 1.13E-42 | 736.34589 |
| rs268124 | 2:65654364 | T | C | 0.18633 | 0.2744 | 0.03237 | 8.60E-09 | 194.49236 |
| rs34703115^#^ | 2:40282854 | C | T | -0.61619 | 0.0328 | 0.10478 | 4.08E-09 | 335.37073 |
| rs35000415^*^ | 7:128585616 | T | C | 0.58779 | 0.8996 | 0.04154 | 1.86E-45 | 834.71443 |
| rs35251378^*^ | 19:10475652 | A | G | -0.23572 | 0.2694 | 0.03243 | 3.61E-13 | 305.19297 |
| rs353608 | 11:35101738 | G | A | 0.18633 | 0.5477 | 0.02802 | 2.93E-11 | 241.15772 |
| rs4388254^*^ | 5:133428601 | T | C | 0.37844 | 0.9294 | 0.06040 | 3.71E-10 | 263.05961 |
| rs4916215 | 1:173314540 | T | C | 0.22314 | 0.2545 | 0.03397 | 5.07E-11 | 264.43768 |
| rs58721818 | 6:138243739 | T | C | 0.65752 | 0.9751 | 0.07559 | 3.38E-18 | 293.19296 |
| rs6679677 | 1:114303808 | A | C | 0.33647 | 0.0915 | 0.04649 | 4.55E-13 | 263.44703 |
| rs6889239 | 5:150457771 | C | T | 0.27763 | 0.2575 | 0.03174 | 2.19E-18 | 408.05769 |
| rs7097397^*^ | 10:50025396 | A | G | -0.18633 | 0.3956 | 0.02871 | 8.60E-11 | 232.90410 |
| rs73050535^#^ | 12:5012503 | T | C | -0.71335 | 0.9702 | 0.12413 | 9.11E-09 | 407.39327 |
| rs7768653 | 6:106574794 | T | C | -0.20701 | 0.4016 | 0.02969 | 3.11E-12 | 287.77145 |
| rs9852014^*^ | 3:129084581 | G | A | 0.62058 | 0.9254 | 0.04927 | 2.26E-36 | 718.17811 |

Abbreviations: SLE, systemic lupus erythematosus; PBC, primary biliary cholangitis; SNP, single nucleotide polymorphism; EA, effect allele; OA, other allele; SE, standard error. * Outlier SNPs removed by MR-PRESSO; # Outlier SNPs removed by IVW Radial method.

Table S11. The casual association between SLE and PBC using various methods(Validation cohort).

| **Exposure** | **Outcome** | **Method** | **Nsnp** | **B** | **Se** | **P-value** | **OR** | **LowerCI** | **UowerCI** |
| --- | --- | --- | --- | --- | --- | --- | --- | --- | --- |
| **The preliminary results of the causal relationship between SLE and PBC** | | | | | | | | | |
| SLE | PBC | MR Egger | 23 | 0.00347 | 0.12722 | 0.979 | 1.00347 | 0.78202 | 1.28764 |
| SLE | PBC | Weighted median | 23 | 0.10065 | 0.03877 | 0.009 | 1.10589 | 1.02497 | 1.19320 |
| SLE | PBC | Inverse variance weighted | 23 | 0.20683 | 0.06142 | 0.001 | 1.22978 | 1.09031 | 1.38709 |
| SLE | PBC | Simple mode | 23 | 0.29056 | 0.13745 | 0.046 | 1.33718 | 1.02139 | 1.75059 |
| SLE | PBC | Weighted mode | 23 | 0.00326 | 0.03504 | 0.927 | 1.00326 | 0.93668 | 1.07458 |
| **The ultimate results of the causal relationship between SLE and PBC** | | | | | | | | | |
| SLE | PBC | MR Egger | 13 | 0.13875 | 0.07887 | 1.06E-01 | 1.14883 | 0.98428 | 1.34090 |
| SLE | PBC | Weighted median | 13 | 0.30064 | 0.04255 | 1.60E-12 | 1.35072 | 1.24264 | 1.46820 |
| SLE | PBC | Inverse variance weighted | 13 | 0.24789 | 0.03314 | 7.44E-14 | 1.28132 | 1.20073 | 1.36731 |
| SLE | PBC | Simple mode | 13 | 0.33489 | 0.07063 | 4.79E-04 | 1.39779 | 1.21709 | 1.60531 |
| SLE | PBC | Weighted mode | 13 | 0.32846 | 0.10174 | 7.24E-03 | 1.38883 | 1.13774 | 1.69534 |

Abbreviations: SLE, systemic lupus erythematosus; PBC, primary biliary cholangitis; OR, odds ratio; CI, confidence interval.

Table S12. Sensitivity analysis of MR analyses of SLE on PBC(Validation cohort).

| **Outcome** | **Number of IVs** | **Heterogeneity (IVW)** | | **Heterogeneity (MR-Egger)** | | **Pleiotropy test** | | **Outliers** |
| --- | --- | --- | --- | --- | --- | --- | --- | --- |
|  |  | **Q** | **P-value** | **Q** | **P-value** | **Intercept** | **P-value** |  |
| Primary Outcome | 23 | 256.004 | ＜0.001 | 221.775 | ＜0.001 | 0.079 | 0.086 | None |
| Secondary Outcome | 15 | 33.973 | 0.002 | 23.544 | 0.036 | 0.057 | 0.032 | MR-PRESSO  (rs1078324, rs1464446, rs2573219, rs35000415, rs35251378, rs4388254, rs7097397, rs9852014) |
| Final Outcome | 13 | 17.557 | 0.130 | 14.544 | 0.204 | 0.033 | 0.160 | IVW Radial method (rs34703115, rs73050535) |

Abbreviations: IVs: instrumental variables; IVW, inverse-variance weighted; PRESSO, Pleiotropy Residual Sum and Outlier; MR, Mendelian randomization.

Table S13. Multivariable MR analysis for evaluating effects of BMI, smoking, and alcohol consumption on the results.

| **MVMR** | **Number of SNPs** | **Beta** | **SE** | **P-value** | **OR(95%CI)** |
| --- | --- | --- | --- | --- | --- |
| **SLE(Outcome)** |  |  |  |  |  |
| PBC | 10 | 0.29361 | 0.04116 | 9.76E-13 | 1.34126(1.23731,1.45393) |
| BMI | 5 | -0.16761 | 0.20264 | 0.408 | 0.84568(0.56848,1.25805) |
| Smoking | 8 | 0.08737 | 0.66902 | 0.896 | 1.09130(0.29407,4.04975) |
| Alcohol consumption | 226 | 0.12939 | 0.19191 | 0.500 | 1.13814(0.78133,1.67589) |
| **PBC(Outcome)** |  |  |  |  |  |
| SLE | 12 | 0.18175 | 0.03205 | 1.43E-08 | 1.19932(1.12629,1.27708) |
| BMI | 5 | 0.12599 | 0.18009 | 0.484 | 1.13427(0.79692,1.61400) |
| Smoking | 8 | 0.57166 | 0.62527 | 0.361 | 1.77120(0.52003,6.03266) |
| Alcohol consumption | 220 | 0.19170 | 0.17891 | 0.284 | 1.21130(0.85303,1.72005) |

Abbreviations: MR, Mendelian randomization; OR, odds ratio; CI, confidence interval; SLE, systemic lupus erythematosus; PBC, primary biliary cholangitis; BMI: Body mass index.


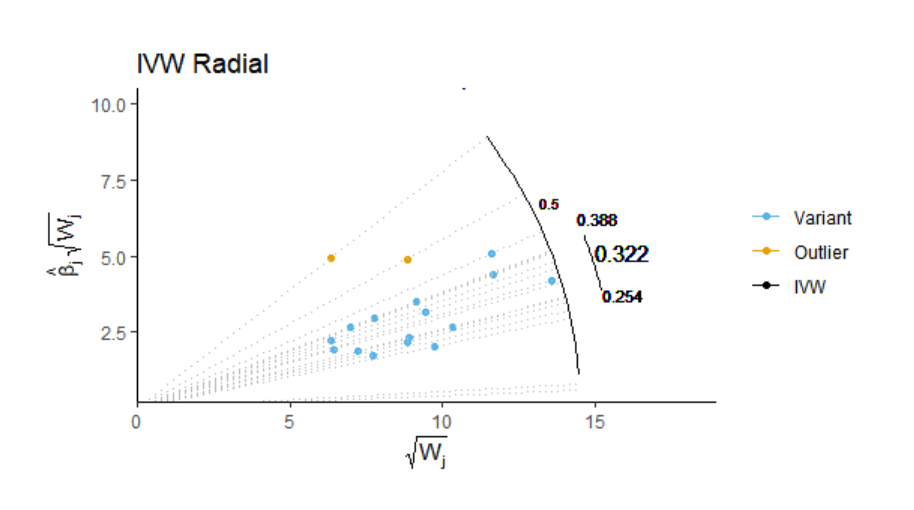


Figure. S1 The radial plot of outliers of PBC on SLE. IVW, inverse-variance weighted.


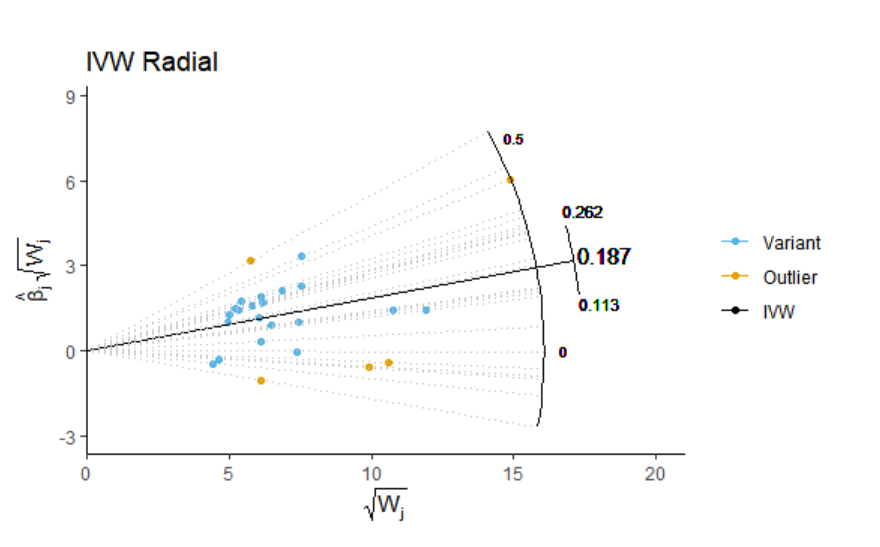


Figure.S2 The Radial plot of outliers of SLE on PBC. IVW, inverse-variance weighted.


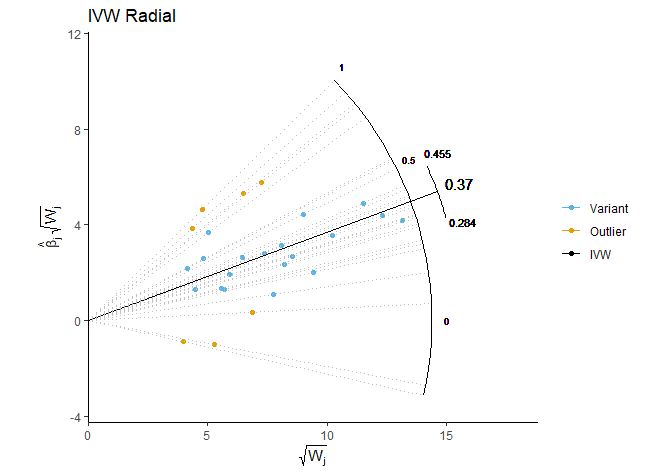


Figure.S3 The Radial plot of outliers of PBC on SLE(Validation cohort). IVW, inverse-variance weighted.


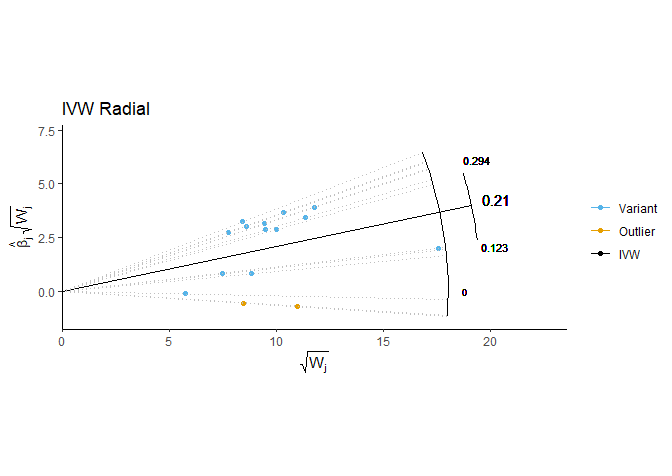


Figure.S4 The Radial plot of outliers of SLE on PBC(Validation cohort). IVW, inverse-variance weighted.


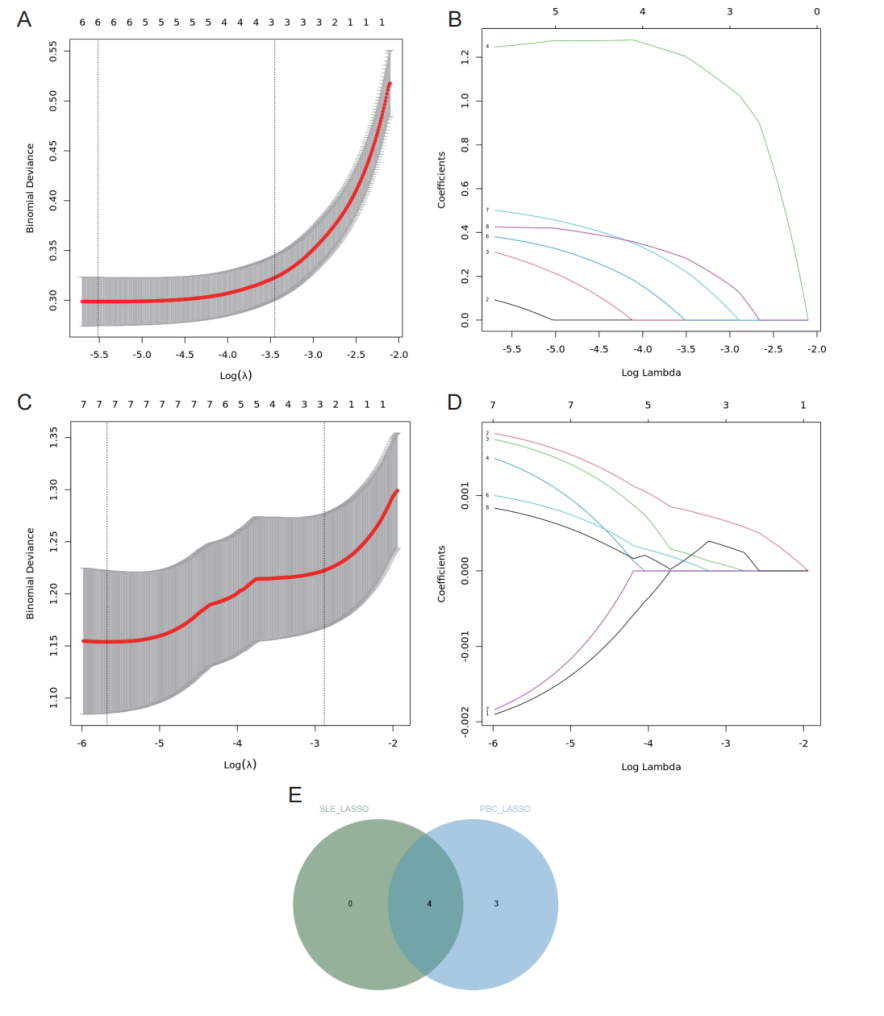


Figure. S5 Identification of potential shared diagnostic genes by the LASSO regression model. (A) Tenfold cross-validation to select the optimal tuning parameter log (lambda) in the GSE65391 database. (B) LASSO coefficient profiles of diagnostic genes in the GSE65391 database. (C) Tenfold cross-validation to select the optimal tuning parameter log (lambda) in the GSE119600 database. (D) LASSO coefficient profiles of diagnostic genes in the GSE119600 database. (E) Venn diagram shows the optimal diagnostic biomarkers.
